# O11.1. A RANDOMISED CONTROLLED TRIAL OF SMARTPHONE ACTIVE SYMPTOM MONITORING IN PSYCHOSIS

**DOI:** 10.1093/schbul/sby015.261

**Published:** 2018-04-01

**Authors:** Shon Lewis, Paolo Fraccaro, John Ainsworth, Matt Machin, Caroline Sanders, Zhimin He, Pauline Whelan, Charlotte Stockton-Powdrell, Richard Hopkins, Til Wykes

**Affiliations:** 1 University of Manchester; 2 Aberystwyth University; 3 King’s College London

## Abstract

**Background:**

We developed a smartphone-based personalised technology to monitor symptoms in real time and showed good acceptability, reliability and validity for active remote monitoring of symptoms in previous published studies (www.clintouch.com). We report a randomised trial testing its efficacy in improving psychotic symptom control, and its potential as an early warning system for relapse when embedded into the ICT systems of mental health provider organisations, and as a tool for identifying new phenotypes for precision medicine.

**Methods:**

Participants with SMI receive a semi-random beep 2–4 times per day on their smartphone app and answer 14 key symptom rating items using a touchscreen slider. Responses are uploaded wirelessly in real time to a central server and build into a graphical readout on the handset, allowing active symptom monitoring and attempts at self-management. We built this into an end-to-end system in two NHS Hospital Trusts (Manchester and South London) to stream data into electronic care records and enable detection by the clinical team of early signs of relapse in people with SMI when key symptoms exceeded a personalised severity threshold. We conducted an open randomised controlled trial of this active symptom monitoring (ASM) using the smartphone app compared to usual management with the aim of assessing: (i) acceptability of continuous monitoring over 3 months; (ii) impact of active self-monitoring on PANSS positive symptoms and Empowerment Rating Scale score assessed at 6 and 12 weeks; (iii) efficiency of detecting early warning signs of relapse. Eligible participants with a DSM5 diagnosis of schizophrenia and related disorders and a history of relapse within the previous two years were included from an early intervention team (early psychosis group) and a community team (chronic psychosis group).

**Results:**

Of 181 eligible, 81 were randomised to either active symptom monitoring or management as usual. 90% stayed in the trial for 12 weeks. Of the 38 in the ASM arm who completed 12-week follow up, adherence defined as responding to >33% of alerts was 84%, >50% of alerts was 60%. At 12 weeks, ASM compared to usual management was associated with no difference on empowerment scale. PANSS positive subscale score showed a significant mean reduction in the ASM group over 12 weeks in the early psychosis group (n= 22, planned ANCOVA p<0.02), but no effect in the chronic psychosis group (n=19). Early warning sign alerts generated by the system occurred in 92% of cases and blind comparison with electronic case record data suggested good sensitivity and lower specificity, but with clear indications of how to adjust the gain of the system to improve future event-detection efficiency. Multivariate analyses pointed to the ability of the system to identify clinical subtypes.

**Discussion:**

The active smartphone monitoring system is feasible and acceptable over three months in people with schizophrenia and related disorders. It was associated with psychotic symptom improvement in recent onset participants, supporting the notion of improved self-management. When built into clinical management workflows to enable personalised alerts of symptom deterioration, it was shown to have potential use in promoting earlier intervention for relapse.

